# Hepatocyte Paraffin 1 Immunoreactivity in Early Colon Carcinogenesis

**DOI:** 10.4021/gr2009.10.1313

**Published:** 2009-09-20

**Authors:** Sonia Nemolato, Alberto Ravarino, Daniela Fanni, Pierpaolo Coni, Eliana Di Felice, Giancarlo Senes, Gavino Faa

**Affiliations:** a Department of Cytomorphlogy, Division of Pathology, University of Cagliari, Cagliari, Italy; b Corrisponding Author: Dipartimento di Citomorfologia, Divisione di Anatomia Patologica, Universita di Cagliari, Via Ospedale 46, 09124 Cagliari, Italy. Email: sonianemolato@libero.it; sonia.nemolato@tiscali.it

**Keywords:** Colorectal polyps, Colorectal adenomas, Colorectal adenocarcinomas, Hepatocyte paraffin 1

## Abstract

**Background:**

This study was aimed at evaluating the correlation between Hepatocyte paraffin 1 (Hep par 1) and colorectal cancer.

**Methods:**

To this end, 50 intestinal biopsies were analyzed including 10 colorectal polyps with low grade dysplasia, 10 with high grade dysplasia, 10 colorectal adenocarcinomas, 10 specimens of normal ileum and 10 of normal colon mucosa. Tissue sections were immunostained for Hep par 1 utilizing a commercial antibody. Normal colonic mucosa did not express Hep par 1.

**Results:**

Immunoreactivity for Hep par 1 was detected in 20% of polyps with low grade dysplasia, 50% of polyps with high grade dysplasia and 60% of colorectal carcinomas. Hep par 1 was frequently detected in the deepest areas of adenocarcinomas mainly in infiltrating tumour cells.

**Conclusions:**

Our data show that Hep par 1 immunoreactivity in human colon carcinogenesis is correlated with progression from low grade to high grade dysplasia and adenocarcinoma. In clinical practice, our data show that caution should be taken in utilizing Hep par 1 as the sole tool in differentiating hepatocellular carcinoma from a liver metastasis of colon adenocarcinoma. Our data encourage further investigations into the potential role played by Hep par 1 in gastrointestinal carcinogenesis.

## Introduction

Hepatocyte paraffin 1 (Hep par 1), is a monoclonal antibody developed in 1993 which recognizes an epitope localized in hepatocyte mitochondria [1], identified as carbamoyl phosphate synthetase 1 (CPS1) [2]. Immunoreactivity for this antibody is generally considered the most specific and sensitive marker of normal and neoplastic hepatocytes and it has been used in the differential diagnosis of hepatocellular carcinoma versus metastatic colorectal carcinoma [1, 3-6].

Hep par 1 immunoexpression is normally cytoplasmic and granular. It is diffuse in trabecular HCC and it is only seen focally in the glandular areas [5, 7, 8]. The intensity of the immunoistochemical reaction seems to be related to the degree of hepatocyte differentiation in hepatoblastoma [8-10].

Recent data also suggest that non hepatic neoplasms might express this marker: Hep par 1 reactivity has been reported in gastric tumours with hepatoid histotype [9-11], and in some cases of pancreas, ovary, breast and neuroendocrine carcinomas [1, 10, 12-16].

Immunostaining for Hep par 1 has been shown to be variable from one case to the next, particularly in gastric neoplastic cells, suggesting a low grade of specificity of this antibody in this type of tumors [17]. Conflicting results have recently been reported on colorectal cancer: Hep par 1 positive cells were found respectively in 4% [14] and in 50% of large bowel carcinomas [17], in 22% of colon signet-ring cell carcinoma [12], and in 2% of colon adenomas with high grade dysplasia. In Barrett esophagus, Hep par 1 was found in a large percentage of cases, leading to suggest it a highly specific immunomarker [18]. Hep par 1 immunoreactivity, normally present in the small intestinal epithelial cells, was found to be absent in a large number of small intestinal adenocarcinomas, suggesting a functional role for the disappearance of this protein during small intestinal tumorigenesis [19].

While these findings obviously seem to reduce the high specificity of Hep par 1 as a diagnostic marker for HCC, they also emphasize the need for a comprehensive early analysis of suggested diagnostic markers in all different types of normal and neoplastic tissues [20]. In this study we analyzed several cases of colorectal adenomas with low grade and high grade dysplasia and multiple cases of colorectal adenocarcinomas in order to evaluate a possible association between Hep par 1 immunorectivity and colorectal carcinogenesis and its progression.

## Materials and Methods

Fifty intestinal biopsies were selected from the medical records and archival slides of our institute. The following intestinal biopsies were analyzed in this study: 10 consecutive colorectal polyps with low grade dysplasia; 10 colorectal polyps with high grade dysplasia; 10 colorectal adenocarcinomas; 10 specimens of normal ileal mucosa and 10 specimens of normal colorectal mucosa. As a positive control, we utilized two human liver needle biopsies. As a negative control, we used 5 normal colon biopsies. All samples had been fixed in 10% formalin, paraffin-embedded and routinely processed. 5 micron-thick sections from each case were immunostained for Hep par 1 (Dako, clone OCH1E5.2.10, 1:80 diluition, Carpintera, CA). All cases were independently reanalyzed by two pathologists specialized in gastrointestinal pathology (SN, GF), according to the 1999 WHO classification. All cases were reviewed with the Hep par 1 immunoreactivity.

## Results

Normal human colonic mucosa did not express Hep par 1 immunoreactivity in all tested cases, while a diffuse granular cytoplasmic immunostaining was observed in all tested liver biopsies (positive control).

**Figure 1:**
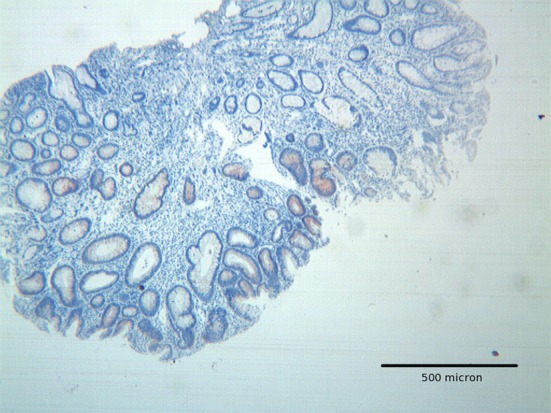
Tubular adenoma with low grade dysplasia, showing focal Hep par 1 positivity mainly located in areas with low grade dysplasia. H&E original magnification x 100.

**Figure 2:**
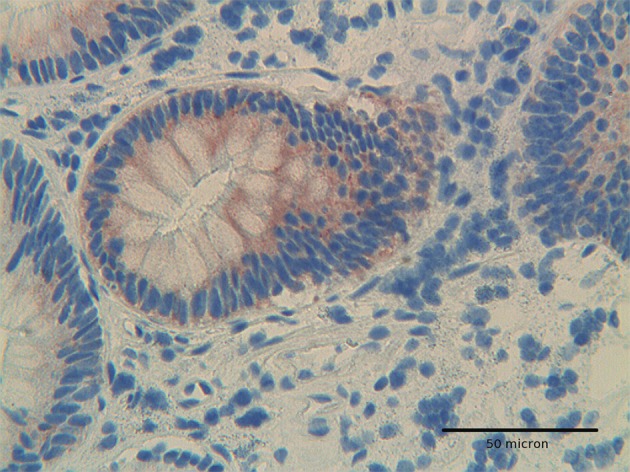
At high power, Hep-Par1 shows a granular cytoplasmic pattern, related to its mitochondrial location, in low grade dysplasia adenoma. H&E original magnification x 400.

**Figure 3:**
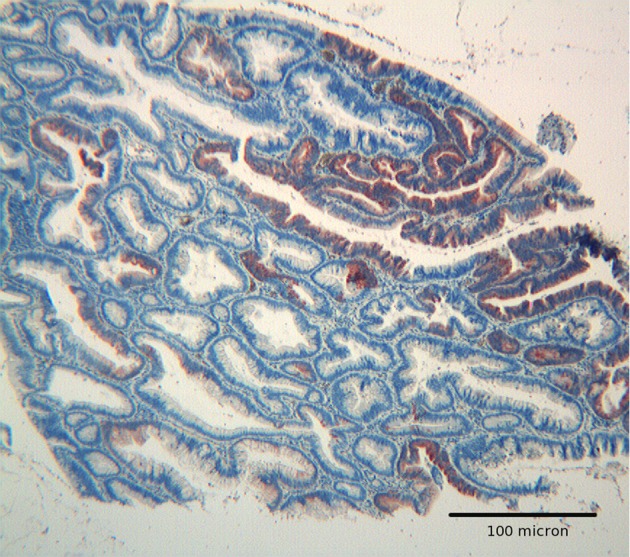
Tubulo villous adenoma with high grade dysplasia. Hep par 1 strong reactivity confined to an area of high grade dysplasia. Mild focal immunoreactivity is also present in glands with low grade dysplasia. H&E original magnification x 250.

**Figure 4:**
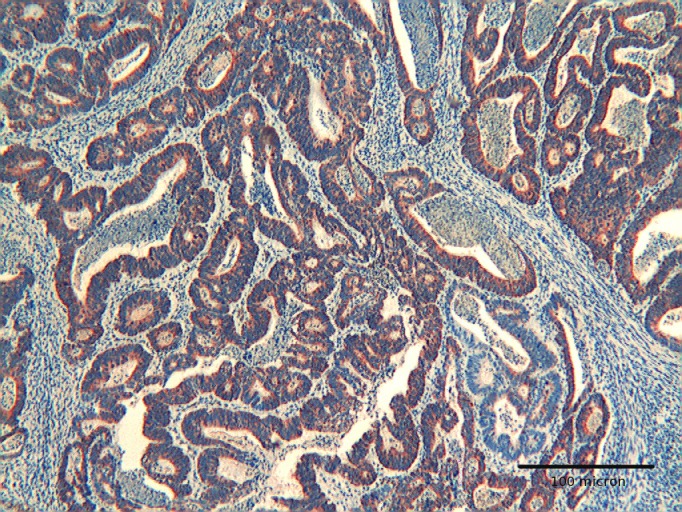
Colon adenocarcinoma showing diffuse immunoreactivity for Hep par 1. H&E original magnification x 250.

**Figure 5:**
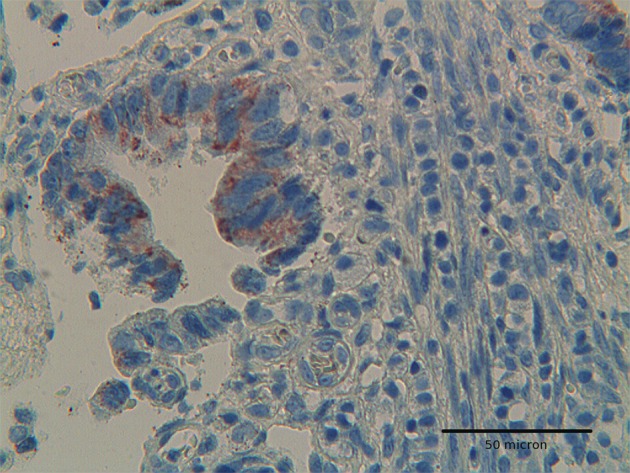
Colon adenocarcinoma showing positivity for Hep-par 1 in deep infiltrating tumor cells. H&E original magnification x 400.

The immunoexpression of Hep par 1 in colorectal polyps and in colorectal adenocarcinomas varied from one case to the next. Immunoreactivity for Hep par 1 was detected in 20 % of polyps with low grade dysplasia (Fig. 1, 2), in 50 % of polyps with high grade dysplasia (Fig. 3) and in 60% of colorectal cancer (Fig. 4, 5). Two main patterns of immunoreactivity were observed in colorectal adenomas. In colorectal adenocarcinomas, immunoreactivity for Hep par 1 changed from granular to diffuse to the entire cytoplasm (Fig. 4, 5). Moreover, Hep par 1 reactivity appeared diffuse to the majority of tumor cells (Fig. 4, 5). Granular cytoplasmic immunoreactivity was interpreted as mitochondrial localization of Hep par 1, while the cytoplasmic diffuse stain was probably due to its dislocation to the cytosol of dysplastic and neoplastic cells. Intratumoral variability in the expression of Hep par 1 was observed in all positive cases, with immunoreactive areas adjacent to negative zones. Moreover, in some cases, a positive trend in the degree of expression from the superficial areas to the deeper regions was observed. The highest degree of immunoreactivity for Hep par 1 was frequently found in infiltrating tumour cells as the deep margins of the tumor (Fig. 5).

Adenomas with low grade dysplasia showed a patchy and focal immunoreactivity, with positive areas surrounded by negative glands. In this setting, hyperplastic glands appeared constantly devoid of immunoreactive cells (Fig. 1). Positive cells were characterized by a granular reactivity in the cytoplasm (Fig. 2). In high grade adenomas we detected a diffuse reactivity for Hep par 1 which appeared stronger in areas with high dysplasia and weak in glands with low dysplasia. The type of cellular reactivity was granular, similar to that found in low grade adenomas (Fig. 3).

## Discussion

The immunoexpression of Hep par 1 in human colon polyps and in colorectal adenocarcinomas has been investigated for some time in previous studies with contradictory results [1, 10, 12-14). Villari et al [17] demonstrated that colon adenocarcinoma cells express Hep par 1 in a percentage close to 50% [17]. On the contrary other authors [14] reported that in colon cancer, Hep par 1 immunoreactivity may be detected in a very low percentage of cases [14], being negative in the majority of colon tumors. Other authors showed that Hep par 1 was expressed also in preneoplastic cells, i.e. in colorectal polyps with dysplasia, possibly representing an early change during human colon carcinogenesis [19, 20]. Recently, it has been reported that CPS1 is overexpressed in approximately 43% of human colon cancer, suggesting a role for Hep par 1 in the progression of colorectal adenocacinoma [19].

To the best of our knowledge, it has not yet been clarified which subset of tumour cells first acquire Hep par 1 expression. In particular, since human colonic mucosa does not express Hep par 1 at all, as confirmed in our study, it has not been clarified which role could be play by Hep par 1 in the progression from low grade dysplasia towards colon cancer. In this study, we provide immunohistochemical evidence that Hep par 1 is expressed not only in the majority of colon adenocarcinomas but also in colon polyps with mild dysplasia as well as in a percentage of polyps with severe dysplasia. Moreover, in adenomas Hep par 1 was constantly not expressed in hyperplastic glands, while its expression was mainly found in dysplastic glands, supporting the hypothesis of an active role for Hep par 1 in colon cancer insurgence. The detection of Hep par 1 immunoreactivity in a higher percentage of polyps with high dysplasia, as compared with polyps with low dysplasia, suggests a possible role for Hep par 1 in colon cancer progression. The localization of Hep par 1 in mitochondria (appearing as immunoreactive cytoplasmatic granules) or in the cytosol (appearing as a diffuse cytoplasmic stain) may be indicative for a biological role of colon neoplastic cell in the production of CPS1. The new finding of a prevalent Hep par 1 immunoexpression in the deepest areas of adenocarcinomas, mainly in infiltrating tumour cells, strongly supports the hypothesis that Hep par 1 could play some role not only in cancer insurgence and progression, but also in invasion. Recently, changes in other mitochondrial proteins have been reported in human colon carcinogenesis. Uncoupling protein-2 (UCP2) an anion carrier located in the inner membrane of mitochondria, has been shown to be overexpressed in colon tubular adenomas and in most colorectal adencarcinomas [21]. Moreover, changes in mitochondrial cytochrome oxidase subunit 3 (COIII) have been found during colon carcinogenesis, with a progressive decrease from normal mucosa to adenomas and carcinomas [22]. Cytochrome oxidase subunit 1 (COI) expression has been found to be different in colon carcinomas according to the degree of differentiation, being higher in well-differentiated carcinomas as compared to those which are poorly differentiated [23].

Taken together, our findings indicate that Hep par 1 could play some role in human colon carcinogenesis. This opens up an interesting perspective for the possible use of Hep par 1 as a marker of progression and transformation into colon cancer. In particular, we may speculate that immunoreactivity for Hep par 1 could help in the identification of a subset of colon polyps with more advanced tumour progression. From a practical point of view, in clinical practice, caution should be taken in utilizing Hep par 1 immunoreactivity as the only tool in differentiating HCC with an adenoid pattern from a liver metastasis from colon cancer, given the possibility of a strong Hep par 1 expression even in colon adenocarcinoma as demonstrated in this study. Our preliminary results should encourage further investigation into the potential role that Hep par 1 expression could play in gastrointestinal pathobiology and carcinogenesis.
